# A Simplified Iohexol-Based Method to Measure Renal Function in Sheep Models of Renal Disease

**DOI:** 10.3390/biology9090259

**Published:** 2020-08-31

**Authors:** Sergio Luis-Lima, Carolina Mas-Sanmartin, Ana Elena Rodríguez-Rodríguez, Esteban Porrini, Alberto Ortiz, Flavio Gaspari, Laura Diaz-Martin, Anders Åsberg, Trond Jenssen, Alejandro Jiménez-Sosa, Paula Martinez-Ros, Antonio Gonzalez-Bulnes

**Affiliations:** 1 Department of Nephrology and Hypertension, IIS-Fundacion Jimenez Diaz, 28040 Madrid, Spain; sergio.lima@quironsalud.es (S.L.-L.); AOrtiz@fjd.es (A.O.); 2 Dpto. de Produccion y Sanidad Animal, Facultad de Veterinaria, Universidad Cardenal Herrera-CEU, CEU Universities, C/Tirant lo Blanc, 7, 46115 Alfara del Patriarca Valencia, Spain; carolina.mas@alumnos.uchceu.es (C.M.-S.); paula.martinez@uchceu.es (P.M.-R.); 3 Research Unit, Hospital Universitario de Canarias, 38320 La Laguna, Tenerife, Spain; anarrguez@gmail.com (A.E.R.-R.); lauradiazmart@gmail.com (L.D.-M.); ajimenezsosa@gmail.com (A.J.-S.); 4 Internal Medicine Department, Hospital Universitario de Canarias, 38320 La Laguna, Tenerife, Spain; esteban.l.porrini@gmail.com; 5 Laboratorio Función Renal, Universidad de La Laguna, 38320 La Laguna, Tenerife, Spain; flagas52@gmail.com; 6 Department of Pharmacy, University of Oslo, 0316 Oslo, Norway; anders.asberg@farmasi.uio.no; 7 Department of Transplantation Medicine, Oslo University Hospital Rikshospitalet, 0424 Oslo, Norway; trond.jenssen@ous-hf.no; 8 Instituto Nacional de Investigación y Tecnología Agraria y Alimentaria (INIA), Avda Pta. de Hierro s/n, 28040 Madrid, Spain

**Keywords:** renal function, iohexol plasma clearance, sheep model

## Abstract

Sheep are highly adequate models for human renal diseases because of their many similarities in the histology and physiology of kidney and pathogenesis of kidney diseases. However, the lack of a simple method to measure glomerular filtration rate (GFR) limits its use as a model of renal diseases. Hence, we aimed to develop a simple method to measure GFR based on the plasma clearance of iohexol by assessing different pharmacokinetic models: (a) CL2: two-compartment (samples from 15 to 420 min; reference method); (b) CL1: one-compartment (samples from 60 to 420 min); (c) CLl*f*: CL1 adjusted by a correction formula and (d) SM: simplified CL2 (15 to 300 min). Specific statistics of agreement were used to test the models against CL2. The agreement between CL1 and CL2 was low, but both CL1*f* and SM showed excellent agreement with CL2, as indicated by a total deviation index of ~5–6%, a concordance correlation of 0.98–0.99% and a coverage probability of 99–100%, respectively. Hence, the SM approach is preferable due to a reduced number of samples and shorter duration of the procedure; two points that improve animal management and welfare.

## 1. Introduction

Preclinical research on the pathogenesis and treatment of renal disease is largely sustained on translational studies in animal models. Rodents, mainly mice and rats, are the species generally preferred in preclinical studies, because of the benefits related to cost, short lifecycle and the availability of genetically modified strains [1,2]. These models are useful for analyzing molecular aspects of renal damage. However, there are profound differences between mice and humans in renal anatomy (i.e., single papilla, undivided medulla and cortex, physiology and pathogenesis of renal disease), lowering the reliability of small animal models for translational renal research [3,4,5,6].

Hence, there is also a profuse use of large animal models (i.e., sheep and pig [7]) for the study of renal diseases. The advantages of these models are multiple. First, from anatomical and physiological perspectives, the multipapillary kidney of sheep and pig has more similarities with humans than any other species except non-human primates. In fact, the only difference among human, swine and sheep kidneys is that the collecting ducts of sheep consist only of a renal pelvis with several recesses [8]. Second, the body size of large animals allows the use of imaging techniques and serial sampling of large amounts of blood and tissues. Third, the longer life cycle of large animals allows long-term studies. Finally, housing and management are easier and less questionable than in primates or carnivores. In consequence, pig and sheep have been frequently used for research in renal diseases [9,10,11,12,13,14,15,16,17,18]. Sheep have additional advantages to pigs, like lower costs (purchasing, housing and feeding), more affordable regulations for housing and handling waste removal, a calm nature which makes them easy to manage and reduces ethical implications and, finally, hemodynamic and coagulation systems which are similar to humans, and simple anatomical features of the neck which facilitate vascular access [10].

However, a major limitation for the use of sheep as a model of renal disease is the lack of a simple and reliable method to evaluate glomerular filtration rate (GFR), as available in other species. Several methods have been described to measure GFR, including the clearance of inulin, radioactively labeled markers such as ^51^Cr–EDTA, ^125^I–iothalamate, and ^99^mTc–DTPA (diethylene-triamine-pentaacetate), or non-radioactive markers such as iohexol and iothalamate [19,20]. The plasma clearance of iohexol is currently accepted as a “practical” gold-standard, since it is simple, reliable and safe even in patients with advanced chronic renal disease (CKD; [19,20,21]). Moreover, the agreement with other methods, in particular with the clearance of inulin (a more complex method), is excellent [22]. However, to the best of our knowledge, there is only one study on the clearance of iohexol in sheep [23]. In such a study, two iohexol-based methods using 13 or 9 samples depending on the pharmacokinetic model used (two- or one-compartments models) were compared in conscious sheep. However, the method has some main limiting factors related to animal-welfare (high number of samples needed and excessive length of sampling period).

Taking into consideration the 3Rs model for animal research (refinement, reduction and replacement; [24]), there is a need for a simplified method for GFR evaluation, like previously reported for pigs [25]. We aimed to develop a simple and reliable method to measure GFR using the plasma clearance of iohexol, with a reduced number of blood samples and/or a shorter time for the procedure in conscious and unrestrained sheep. In agreement with previous studies in humans [26], we addressed three specific objectives. First, we tested the appropriateness of the one- and two-compartment model previously described for determining iohexol disappearance from the blood and therefore GFR in sheep [23]. Second, we examined whether a one-compartment model adjusted by a formula or a new pharmacokinetic approach using a simplified two-compartment model with a few samples from both the phases of distribution and elimination yield a satisfactory determination of GFR. Third, to further check the accuracy and precision of the simplified method, we applied the same approach on available data from a previous publication of our group in swine [25].

## 2. Material and Methods

### 2.1. Ethics Statement

The experiment was performed in agreement with the Spanish Policy for Animal Protection RD53/2013 and the European Union Directive 2010/63/UE, about the protection of research animals. The experiment was specifically approved by the Universidad Cardenal Herrera Committee of Ethics in Animal Research (report CEEA 18-016) and subsequently by the regional competent authority (2018/VSC/PEA/0197). The sheep were housed at the animal facilities of the Universidad Cardenal Herrera, which meet local, national and European requirements for Scientific Procedure Establishments.

### 2.2. Animals and Experimental Design

The study involved 15 Segureña sheep (8 males and 7 female), 2 to 6 years old and around 50 kg of body weight. The animals were restrained, without sedation or anesthesia, only for sampling during 2–3 min and were free to move during the experimental procedure.

At 8:00 a.m., after 16h fasting, a single dose of 5 mL Omnipaque 300 (GE Healthcare, Madrid, Spain) containing 3.2 g iohexol was injected over 2 min through the jugular vein. We selected a single dose of 5 mL, since it is the same dose used in humans [19,20].

The sampling protocol was based on a previous publication in swine [26]. We assumed a priori a two-compartment model of pharmacokinetic analysis, considering the similarities between renal function and physiology between swine and sheep, and according to Nesje and coworkers [23]. Hence, 5 mL of blood were collected in EDTA vacuum tubes at 15, 30, 45, 60, 90, 120, 180, 240, 300, 360 and 420 min after injection, with a blank (iohexol-free) blood sample being collected before the marker was administered (time zero at 0 min). Blood samples were immediately centrifuged at 3500 rpm for 10 min and plasma was stored at −20 °C until analysis.

### 2.3. Iohexol Measurements

**Figure 1:**
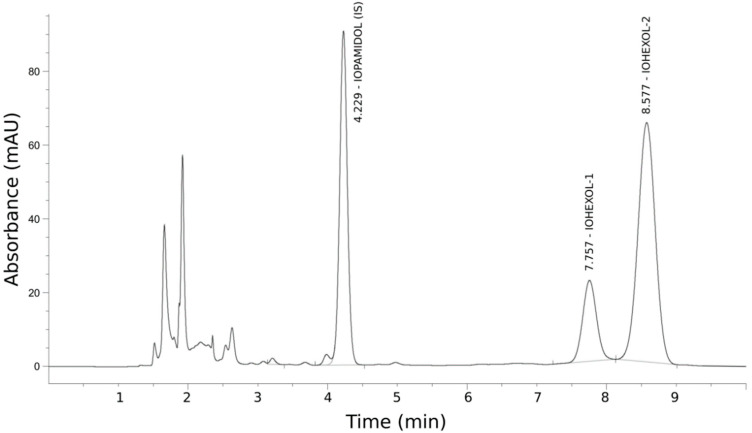
HPLC–UV chromatogram of plasma sample 120 min after iohexol administration.

Iohexol plasma concentrations were measured by HPLC–UV, based on the method described by Krútzen et al. [27] (Figure 1) and adapted after an internal validation (interrater agreement and intrarater precision), and an external validation with the Mario Negri Institute of Italy as reference laboratory [28]. In brief, 200 µL of plasma were added to 50 µL of the internal standard (IS) iopamidol (755 µg/mL) and deproteinized with 750 µL of 5% perchloric acid. Samples were vortexed and centrifuged for 5 min at 12,500 rpm. A 5 µL aliquot of supernatant was chromatographed by a C18 reversed-phase column (5 mm. 150 × 4.6 mm). Advanced Chromatography Technologies Ltd., Aberdeen, UK), using an HPLC system (Agilent Series 1260 Infinity, Santa Clara, CA, USA), equipped with a diode array detector set at 254 nm. Iohexol isomers were eluted by a mixture of deionized water/acetonitrile (96:4 by volume, adjusted to pH 2.5 with phosphoric acid) pumped at a flow rate of 1.0 mL/min. The calculation of the concentrations of iohexol was performed by comparing the height of the peak of the second isomer of iohexol with the IS peak (peak height ratio).

### 2.4. Calibration and Quality Control Standards

Internal calibration curves of iohexol were prepared for each set of samples. A working solution of iohexol (647 µg/mL) was prepared in deionized water and used for the calibration curve and quality control samples. A total of five concentrations of iohexol diluted in plasma were used as calibrators (32.3, 64.7, 97.0, 129.4 and 161.7 µg/mL). Two quality control standards (QCs) were prepared in-house, containing iohexol at low (64.7 µg/mL) and high (129.4 µg/mL) concentrations, and used for validation tests. All the calibrators, quality control samples and reference standard solutions were stored at room temperature until use.

### 2.5. Pharmacokinetic Analyses: One- and Two-compartment Models

**Figure 2:**
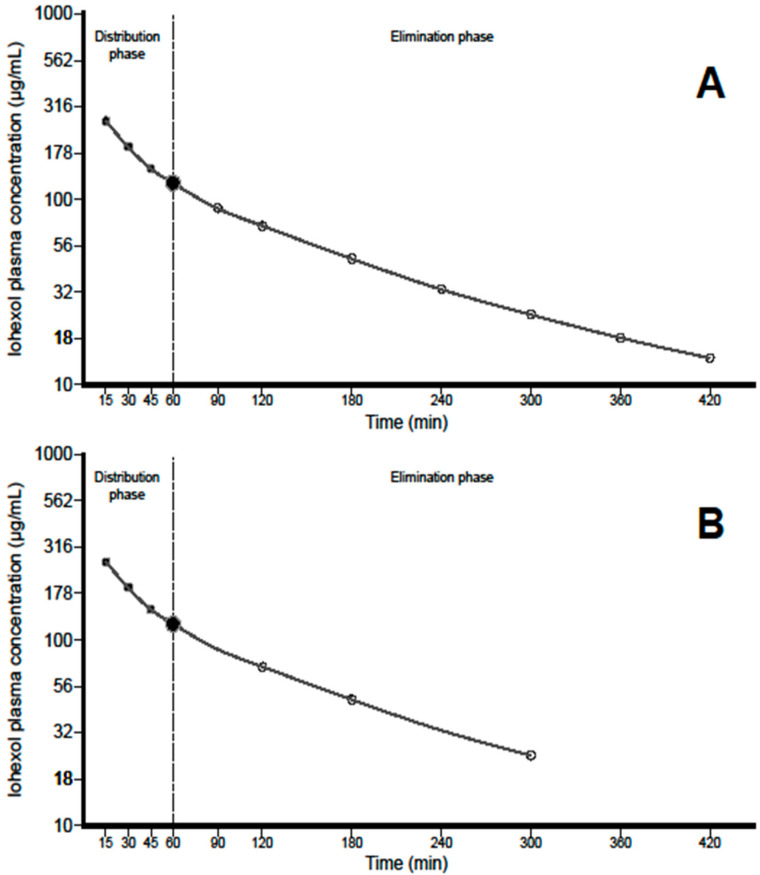
Pharmacokinetic models of the plasma clearance of iohexol in sheep. (**A**) *Two-compartment model* (CL2): includes sampling points of the distribution phase at 15, 30 and 45 min (black circles), the point at 60 min and those of the elimination phase at 90, 120, 180, 240, 300, 360 and 420 min (white circles). The one-compartment model, either with (CL1*f*) or without a formula (CL1), considers only points obtained from 60 min onwards. (**B**) *Simplified method* (SM): a two-compartment model using seven samples from both the distribution and elimination phases (15, 30, 45, 60, 120, 180 and 300 min).

We calculated the plasma clearance of iohexol using 4 different approaches (Figure 2): (a) a two-compartment model, which was considered the reference method (CL2), analyzing a complete pharmacokinetic profile, using points both from the distribution and elimination phases; (b) a one-compartment model (CL1), using only samples from the elimination phase as in a previous publication in sheep [23]; (c) a one-compartment model adjusted by a formula (CL1*f*), which corrects for the AUC not considered in the initial distribution phase; and (d) a simplified two-compartment model (SM), using only a reduced number of samples from both phases.

*a. Two-compartment model* (CL2): The plasma profiles were analyzed by a two-compartment model system. We evaluated both the distribution and elimination phases for a long period of time (420 min), in which samples were taken to determine iohexol at 15, 30, 45, 60, 90, 120, 180, 240, 300, 360 and 420 min (Figure 2A). The area under the curve from 0 420 (AUC_0–420_) was determined by using the trapezoidal rule, whilst the estimated AUC from 420 min to infinity (AUC_420–inf_) was obtained by dividing the concentration of iohexol at 420 min by the elimination constant determined by a log-linear regression of the concentrations from 0 to 420 min. Afterwards, the total AUC from 0 to infinity (AUC_0–inf_) was also calculated. The plasma clearance of iohexol was calculated by the formula: CL = Dose/AUCinf.

*b. One-compartment model* (CL1): The plasma profiles were analyzed by a one-compartment open-model system in which only the elimination phase was assessed by using sampling points from 60 min onwards (i.e., 90, 120, 180, 240, 300, 360 and 420 min). The AUC from 0 to 420 min (AUC_0–420_) was obtained by using the trapezoidal rule, and the area from the last sampling point (420 min) was added by dividing this concentration by the elimination rate constant obtained by a log-linear regression of the concentrations from 60 to 420 min. Therefore, the total AUC from 0 to infinity (AUC_0–inf_) was also determined. The plasma clearance of iohexol was calculated by the formula: CL = Dose/AUC, where AUC is the area under the plasma concentration-time profile.

*c. One-compartment model adjusted by a formula* (CL1f): The use of samples of the elimination phase leads to and underestimation of the area under the curve and, in consequence, to an overestimation of GFR. Thus, a mathematical correction is needed to recalculate the true clearance. For this purpose, we aimed to develop a correction formula which was set up by fitting CL2 plotted against CL1 values and using different equations (linear, quadratic and exponential). For this analysis, the experimental group was divided in two groups of similar characteristics (testing group, *n* = 8, and validation group, *n* = 7).

*d. Simplified two-compartment model* (SM): Samples in both the distribution and elimination phases were selected to reduce both the time of the method and the number of samplings without losing accuracy and precision. According to both findings of Nesje and coworkers [23] and our experimental data, we considered the sampling at 60 min as the pivotal point to separate distribution from elimination phase. Thus, with the aim to simplify as much as possible the sampling schedule and to maintain an adequate number of samples to allow reliable fittings of the data, we selected 3 points per phase (in addition to sampling point at 60 min) as the minimum number of points eligible. As a consequence, the SM model consisted of seven points describing a two-compartment iohexol profile (15, 30, 45, 60, 120, 180 and 300 min). Plasma clearance of iohexol was calculated as Dose/AUC, as described above for the two-compartment model.

### 2.6. Sensitivity Analysis

The measurement of GFR using points from both the distribution and the elimination phases has been scarcely tested in humans [29,30], whilst, to the best of our knowledge, no evidence of its use is available in large animal models. Thus, there is limited evidence of the reliability of this approach so, to further check the accuracy and precision of the simplified method, we applied the same approach on available data from a previous publication of our group in swine [25]. In swine, the elimination phase started at 120 min, and so we selected the following points for the SM approach: 5, 30, 90, 120, 180, 240 and 300 min. The agreement between CL2, CL1f for swine and SM was tested as indicated below in point 2.8.

### 2.7. Statistical Analysis: Tests of Agreement

The agreement between CL2 (reference method) with CL1 CL1*f* and SM was assessed by the limits of agreement described by Bland and Altman [30] and the total deviation index (TDI), concordance correlation coefficient (CCC), and coverage probability (cp), as proposed by Lin et al. [31] The limits of agreement constituted a simple graphic tool which describes the limits that include the majority of the differences between two measurements. The narrower these limits, the better the agreement. CCC combines elements of accuracy and precision. Its scores range from 0 to 1 and a value > 0.90 reflects optimal concordance between measurements. TDI is a measure that captures a large proportion of data within a boundary for allowed differences between two measurements [32]. CP ranges from 0 to 1; it is a statistic that estimates whether a given TDI is less than a pre-specified percentage [33]. The ideal situation is to have a TDI < 10%, meaning that 90% of the estimations fall within an error of ±10% from the gold standard. Finally, these statistics provide confidence intervals that allow the generalization of the results. For the Bland and Altman test, we used the MedCalc statistical software, version 15.8 (MedCalc Software Ltd., Ostend, Belgium). For the agreement analyses, we used the statistical package AGP version 1.0 (Agreement Program, IGEKO. SP; available at: http://lfr.ecihucan.es/apps/agreement_installer/Agreement_Installer.exe), which is based on the R code originally developed by Lin and Yue [33]. The AGP was developed to simplify the use of the tool given in the R agreement package (https://www.R-project.org/.5). Calculations and a graphical representation were performed with SPSS Statistics for Windows, version 17.0 (SPSS Inc., Chicago, IL, USA) and GraphPad Prim 8 software (GraphPad Software, San Diego, CA, USA).

## 3. Results

### 3.1. Iohexol Plasma Analysis

Figure 1 shows an HPLC–UV chromatogram at 120 min after iohexol administration. Iohexol eluted from the chromatographic column as two peaks at 7.8 and 8.6 min, reflecting the isomers present in the pharmacologic preparation. The internal standard iopamidol eluted at 4.2 min. No interfering peaks were observed in iohexol-free samples.

### 3.2. Pharmacokinetic Clearance Profiles

Figure 2A shows the complete two-compartment model (CL2) for the iohexol plasma clearance, which includes a total of 11 sampling points. The first part, up to 60 min, is curvilinear, and corresponds to the distribution phase as observed by Nesje and coworkers [23]. The second part, from 60 to 480 min, is linear, and corresponds to the elimination phase. Accordingly, 60 min was selected as the starting point of the elimination phase. All the 9 sampling points from 60 to 420 min (60, 90, 120, 180, 240, 300, 360 and 420 min) represent the method considered for one-compartment model with (CL1*f*) or without the adjustment formula (CL1).

Figure 2B shows a two-compartment model for the proposed simplified method using only seven sampling points (15, 30, 45 and 60 min for the first part, distribution phase, and 60, 120, 180 and 300 min for the second part of the curve, elimination phase).

### 3.3. Two-Compartment: CL2

Mean GFR was 118 ± 29 mL/min. Individual GFR values are shown in Table 1.

### 3.4. One-Compartment Model: CL1

Mean GFR value was 132 ± 36 mL/min (median 121, IQR: 110–133). In most of the cases, GFR values determined by CL1 were 10–15% higher than those measured with CL2.

### 3.5. One-Compartment Model Adjusted by a Formula: CL1f

Among the several equations tested to develop a correction formula, the best fit with the data was achieved with the quadratic equation: GFR_eq_ = 1.009736 × CL1 − 0.00081765 × CL1^2^, (r^2^ = 0.98). Applying this equation to calculate GFR in all animals, the mean value was 118 ± 27 mL/min. The MAPE was less than 6% for all cases (Table 1).

### 3.6. Simplified Two-Compartment Model (SM)

Mean GFR values were 117 ± 30 mL/min for SM, which was similar to the values obtained by CL2, giving a mean absolute percentage error of 2.6% (Table 1). Individual GFR values are shown in Table 1.

### 3.7. Analysis of Agreement

**Figure 3:**
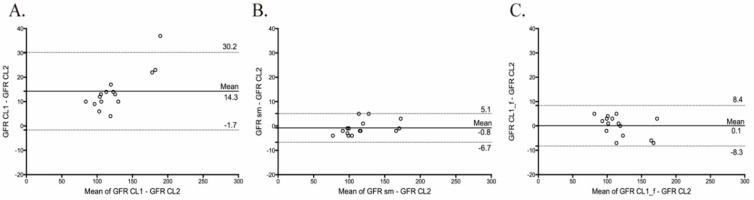
Limits of agreement and Bland–Altman plots for glomerular filtration rate measured (GFR) between the two-compartment model (CL2) with (**A**), the one-compartment (CL1), (**B**) the simplified two-compartment model (SM) and (**C**) the one-compartment adjusted by a formula (CL1*f*). The procedure compares the difference between two methods versus the difference of the means of both methods. The straight and dashed lines indicate mean difference and 95% limits of agreement, respectively.

*a. One-compartment model* (CL1): *Two-compartment (CL2)* vs. *one-compartment model* (CL1): The Bland-Altman plot between the two-compartment (CL2) and one-compartment (CL1) models showed wide limits of agreement (from 30.2 to −1.7 mL/min; Table 2) and a mean difference of 14.3 mL/min, indicating poor agreement (Figure 3A). The concordance correlation coefficient (CCC) between CL1 and CL2 was 0.887% (0.795, upper confidence interval—CI), reflecting low precision and accuracy. The total deviation index (TDI) was 21.7% (26.3, upper CI), indicating that the GFR values measured with CL1 showed an error ranging from about −22 to +22% when compared with the reference method CL2. Finally, the coverage probability (cp) was 36.1 (20.3, upper CI), which indicates that 64% of the GFR values obtained with CL1 had an error range greater than ±10% of those calculated with CL2.

*b. Two-compartment (CL2)* vs. *the one-compartment model adjusted by a formula* (CL1f): The Bland–Altman plot between CL2 and the CL1*f* method showed narrow limits of agreement (from 8.4 to −8.3 mL/min; Table 2) and a mean difference of 0.1 mL/min, indicating an excellent agreement between both approaches (Figure 3C). The CCC between CL2 and CL1*f* was 0.987 (0.971, upper CI), reflects high precision and accuracy. Moreover, the TDI was 6.12 (8.54, upper CI), indicating that 90% of the GFR values with CL1*f* showed an error ranging from −6 to +6%, when compared with the reference method. Finally, the cp was 98.6 (87.1, upper CI), which indicates that almost none of the GFR values determined with the CL1*f* had an error range greater than ±10% of GFR calculated with CL2.

*c. Two-compartment* (CL2) vs. *the simplified two-compartment model* (SM)*:* The Bland–Altman plot between CL2 and the SM showed narrow limits of agreement (from 5.1 to −6.7 mL/min; Table 2) and a mean difference of −0.8 mL/min, indicating excellent agreement between both approaches (Figure 3B). The CCC between CL2 and SM was 0.992 (0.983, upper CI), reflecting both high precision and accuracy. Furthermore, TDI was 4.95% (6.88, upper CI), indicating that 90% of the GFR values with SM showed an error ranging from −5 to +5%, when compared with the reference method. Finally, the cp was 99.8 (93.6, upper CI), which indicates that almost none of the GFR values determined with the SM had an error range greater than ±10% of GFR calculated with CL2.

### 3.8. Sensitivity Analysis

A recalculation of previous data in swine [25] showed that GFR measured with the SM approach was more precise and accurate than the standard procedure using the one-compartment model adjusted by a correction formula. The results in detail are shown in the supplementary material (Supplementary Materials).

## 4. Discussion

We developed a simple and reliable method to assess glomerular filtration rate (GFR) in conscious sheep, by means of the plasma clearance of iohexol. Moreover, we tested a new pharmacokinetic approach using few samples from both the distribution and the elimination phases, which proved to have excellent agreement with the procedure of two-compartment. The method proposed includes the following steps: (i) administration of a single dose of 5 mL of iohexol solution (OMNIPAQUE 300) through an intravenous catheter placed a vein of the neck; (ii) collection of seven blood samples of 5 mL of total blood at 15, 30, 45, 60, 120, 180 and 300 min after injection from a different vein; (iii) determination of iohexol in plasma by HPLC-UV; (iv) fitting the concentrations by double exponential analysis following a two-compartment model and nonlinear regression analysis; and (v) calculation of the area under the curve (AUC) from time zero to infinity, by means of the trapezoidal rule.

The complete two-compartment model, CL2, is the reference method to measure GFR. However, it requires 11 extractions of blood, which makes this approach impractical and not in line with the 3Rs model for animal research (refinement, reduction and replacement). The CL1 and CL1*f* model require less samples i.e., 8 during a period of 420 min and SM uses 7 points over 300 min. The agreement between CL1 and CL2 was not acceptable as indicated by a TDI of 22%, a CCC of 0.89, a cp of 36% and wide limits of agreement. Our results are in line with Nesje and coworkers [23], who found a 10–15% overestimation of GFR with the CL1 method. On the other hand, the CL1*f* model showed excellent agreement with CL2. Finally, the SM also showed an excellent agreement with CL2, as indicated by a TDI of 5%, a CCC of 0.99, a cp of 100% and very narrow limits of agreement. This may be explained by the pharmacokinetic profile of iohexol in sheep, with a more limited distribution phase (i.e., 60 min or less), that accounts only for a small part of the AUC (i.e., 10–15% of the total area). Clearly, both methods can be used to measure GFR, but SM has some advantages compared to CL1*f*, like a reduced number of samples (7 vs. 11) and a shorter duration (300 vs. 420 min). Finally, collecting samples that cover both the distribution and elimination phases of iohexol is more reliable from a pharmacokinetic point of view. Thus, we may consider that SM can be used to measure GFR in sheep models of renal disease.

To our knowledge, there is only one study that evaluated the pharmacokinetics of iohexol in sheep. Nesje and coworkers considered either a one- or two-compartment analysis [23]. This report is highly relevant, since it proved the usefulness and reliability of an iohexol-based method in sheep using 9 samples for the CL1 and 13 for the CL2 method, both during 300 min. As in our study, Nesje and coworkers pointed out a lack of agreement between CL1 and CL2. However, the recommended approach requires 13 samples, which makes the procedure cumbersome. The SM also follows a two-compartment model but requires only half the samples and had an excellent agreement with the longer CL2 approach.

In the standard procedure in humans and swine [25], only samples from the elimination phase are used, and the distribution phase is estimated by a correction formula. In 2006, Schwartz and coworkers [29] proposed a simplification of the protocol in humans by using four plasma samples, including those from the distribution (0–120 min) and elimination phases (from 120 min onwards). The comparison with the reference method using 9 points showed narrow limits of agreement. This study proved, for the first time, that using samples from the first hours was feasible, and that this could help in reducing the duration of the test. Recently, Åsberg and coworkers [30] reproduced the combined use of samples from both the distribution and elimination phase of iohexol in adults and children, showing excellent agreement between the simplified and reference methods (TDI: 7.3%). Based on these data, we decided to analyze the feasibility of adding samples from the distribution phase to those of the elimination phase, in order to reduce the time of the procedure using a limited number of samples. The agreement between the simplified (SM) and the reference method (CL2) was excellent, as indicated above. Moreover, we tested the same procedures using data of our previous study in swine [25], and found an excellent agreement between CL2 and SM methods. Hence, the SM approach is feasible and reliable across different species.

Thus, the plasma clearance of iohexol can be determined using a limited number of samples from both the distribution and elimination phases without losing precision and accuracy. This represents a major simplification of the procedure, and further testing of this approach in other large animal models and humans is worth investigating in future trials.

## 5. Conclusions

In conclusion, we have developed a reliable two-compartment method to measure renal function in sheep. This method is simple, reproducible, accurate and precise, with a reduced number of blood samples, which improves animal management and welfare. Moreover, this simplified method facilitates sequential measurements of renal function, allowing the evaluation of renal function changes over time.

## Figures and Tables

**Table 1:** Iohexol plasma clearance in the one (CL1; CL1*f*) and two-compartment models (CL2). SM = simplified method based on CL2. CL1*f*: CL1 with the adjustment formula. MAPE = mean absolute percentage error.

Sheep	Gender	Weight (kg)	CL2	CL1	CL1*f*	CL SM	MAPE CL2 vs. CL1*f*	MAPE SM vs. CL2
1	F	56	125	135	121	130	3.1	3.3
2	F	49	111	128	116	116	4.2	5.1
3	F	52	171	208	174	170	1.5	0.5
4	F	48	79	89	84	75	5.7	5.0
5	F	40	92	101	94	90	1.8	2.3
6	F	51	99	112	103	96	3.8	3.6
7	F	45	106	120	109	102	3.0	3.7
8	M	45	98	110	101	97	3.2	0.9
9	M	49	101	111	102	97	0.9	4.6
10	M	67	171	194	164	174	4	1.5
11	M	54	117	121	110	115	6	2.2
12	M	65	119	132	119	120	0.2	1.0
13	M	49	100	106	98	99	2.2	1.9
14	M	51	116	130	117	114	1.0	1.6
15	M	104	167	189	161	165	3.7	1.1

**Table 2:** Agreement analysis between GFR values measured with the one-compartment model with (CL1*f*) and without (CL1) an adjustment formula, or the simplified method (SM) with the two-compartment model CL2.

Model	Total Deviation Index (%)	Concordance Correlation Coefficient (%)	Coverage Probability (%)	Limits of Agreement (mL/min)
CL1 vs. CL2	21.7 (26.3)	0.887 (0.795)	36.1 (20.3)	−1.7 to 30.2
CL1*f* vs. CL2	6.12 (8.54)	0.987 (0.971)	98.6 (87.1)	−8.3 to 8.4
SM vs. CL2	4.95 (6.88)	0.992 (0.983)	99.8 (93.6)	−6.7 to 5.1

## Supplementary Materials

The following are available online at https://www.mdpi.com/2079-7737/9/9/259/s1, Table S1: Iohexol plasma clearance in swine. Two compartment model CL2 is the reference method. CL1: one compartment model; CL1f: one compartment model corrected with a formula; SM: simplified two-compartment using 7 points. (MAPE = mean absolute percentage error, Figure S1: Bland–Altman plots of the difference between the glomerular filtration rate (GFR) values measured by the reference method (two-compartment clearance: CL2) and (**A**) the one compartment method adjusted by a formula CL1f and (**B**) the simplified method (SM) versus the differences of the means of both. The straight and dashed lines indicate mean difference and 95% limits of agreement, respectively, Table S2: Agreement between the two-compartment reference method (CL2) with the one-compartment model adjusted by an equation (CL1f) and the two-compartment simplified method (SM) in a group of 16 pigs. TDI: total deviation index, TDI (total deviation index); CCC (concordance correlation coefficient); CP (coverage probability); LA (limits of agreement, mL/min).

## References

[1] Houdebine L.M., Hedrich H. (2004). The mouse as an animal model for human diseases. The Laboratory Mouse.

[2] Muhammad S. (2014). Nephrotoxic nephritis and glomerulonephritis: Animal model versus human disease. Br. J. Biomed. Sci..

[3] Betz B., Conway B.R. (2014). Recent advances in animal models of diabetic nephropathy. Nephron Exp. Nephrol..

[4] Herrmann S.M., Sethi S., Fervenza F.C. (2012). Membranous nephropathy: The start of a paradigm shift. Curr. Opin. Nephrol. Hypertens..

[5] Taylor C.M., Williams J.M., Lote C.J., Howie A.J., Thewles A., Wood J.A., Milford D.V., Raafat F., Chant I., Rose P.E. (1999). A laboratory model of toxin-induced hemolytic uremic syndrome. Kidney Int..

[6] Yokota S.D., Benyajati S., Dantzler W.H. (1985). Comparative aspects of glomerular filtration in vertebrates. Ren. Physiol..

[7] Hamernik D.L. (2019). Farm animals are important biomedical models. Anim. Front..

[8] Buys-Gonçalves G.F., De Souza D.B., Sampaio F.J., Pereira-Sampaio M.A. (2016). Anatomical relationship between the kidney collecting system and the intrarenal arteries in the sheep: Contribution for a new urological model. Anat. Rec..

[9] Becker G.J., Hewitson T.D. (2013). Animal models of chronic kidney disease: Useful but not perfect. Nephrol. Dial. Transplant..

[10] Bujok J., Walski T., Czerski A., Gałecka K., Grzeszczuk-Kuć K., Zawadzki W., Witkiewicz W., Komorowska M. (2018). Sheep model of haemodialysis treatment. Lab. Anim..

[11] Lankadeva Y.R., Kosaka J., Iguchi N., Evans R.G., Booth L.C., Bellomo R., May C.N. (2019). Effects of Fluid Bolus Therapy on Renal Perfusion, Oxygenation, and Function in Early Experimental Septic Kidney Injury. Crit. Care Med..

[12] Alexander Springer A., Kratochwill K., Bergmeister H., Csaicsich D., Huber J., Bilban M., Mayer B., Mühlberger I., Amann G., Horcher E. (2012). A Combined Transcriptome and Bioinformatics Approach to Unilateral Ureteral Obstructive Uropathy in the Fetal Sheep Model. J. Urol..

[13] Lankadeva Y.R., Singh R.R., Tare M., Moritz K.M., Denton K.M. (2014). Loss of a Kidney During Fetal Life: Long-Term Consequences and Lessons Learned. Am. J. Physiol. Renal. Physiol..

[14] Peters C.A. (2001). Animal Models of Fetal Renal Disease. Prenat. Diagn..

[15] Singh R.R., McArdle Z.M., Iudica M., Easton L.K., Booth L.C., May C.N., Parkington H.C., Lombardo P., Head G.A., Lambert G. (2019). Sustained Decrease in Blood Pressure and Reduced Anatomical and Functional Reinnervation of Renal Nerves in Hypertensive Sheep 30 Months After Catheter-Based Renal. Denervation. Hypertension.

[16] Bloor I.D., Sebert S.P., Mahajan R.P., Symonds M.E. (2012). The Influence of Sex on Early Stage Markers of Kidney Dysfunction in Response to Juvenile Obesity. Hypertension.

[17] Vernon K.A., Pickering M.C., Cook T. (2011). Experimental Models of Membranoproliferative Glomerulonephritis, Including Dense Deposit Disease. Contrib. Nephrology.

[18] Connolly F., Rae M.T., Späth K., Boswell L., McNeilly A.S., Duncan W.C. (2015). In an Ovine Model of Polycystic Ovary Syndrome (PCOS) Prenatal Androgens Suppress Female Fetal Renal Gluconeogenesis. PLoS ONE.

[19] Delanaye P., Ebert N., Melsom T., Gaspari F., Mariat C., Cavalier E., Björk J., Christensson A., Nyman U., Porrini E. (2016). Iohexol plasma clearance for measuring glomerular filtration rate in clinical practice and research: A review. Part 1: How to measure glomerular filtration rate with iohexol?. Clin. Kidney J..

[20] Delanaye P., Melsom T., Ebert N., Bäck S.E., Mariat C., Cavalier E., Björk J., Christensson A., Nyman U., Porrini E. (2016). Iohexol plasma clearance for measuring glomerular filtration rate in clinical practice and research: A review. Part 2: Why to measure glomerular filtration rate with iohexol?. Clin. Kidney J..

[21] Donadio C., Tramonti G., Giordani R., Lucchetti A., Calderazzi A., Bassani L., Bianchi C. (1990). Effects on renal hemodynamics and tubular function of the contrast medium iohexol in renal patients. Ren. Fail..

[22] Sterner G., Frennby B., Mansson S., Nyman U., Van Westen D., Almén T. (2008). Determining ’True’ Glomerular Filtration Rate in Healthy Adults Using Infusion of Inulin and Comparing It With Values Obtained Using Other Clearance Techniques or Prediction Equations. Scand. J. Urol. Nephrol..

[23] Nesje M., Flåøyen A., Moe L. (1997). Estimation of Glomerular Filtration Rate in Normal Sheep by the Disappearance of Iohexol From Serum. Vet. Res. Commun..

[24] Russell W.M. (1969). The development of the Animal Care. Lab. Anim. Care.

[25] Luis-Lima S., García-Contreras C., Vázquez-Gómez M., Astiz S., Carrara F., Gaspari F., Negrín-Mena N., Jiménez-Sosa A., Jiménez-Hernández H., González-Bulnes A. (2018). A Simple Method to Measure Renal Function in Swine by the Plasma Clearance of Iohexol. Int. J. Mol. Sci..

[26] Schwartz G.J., Furth S., Cole S.R., Warady B., Muñoz A. (2006). Glomerular filtration rate via plasma iohexol disappearance: Pilot study for chronic kidney disease in children. Kidney Int..

[27] Krutzén E., Bäck S.E., Nilsson-Ehle I., Nilsson-Ehle P. (1984). Plasma Clearance of a New Contrast Agent, Iohexol: A Method for the Assessment of Glomerular Filtration Rate. J. Lab. Clin. Med..

[28] Luis-Lima S., Gaspari F., Porrini E., García-González M., Batista N., Bosa-Ojeda F., Oramas J., Carrara F., González-Posada J.M., Marrero D. (2014). Measurement of glomerular filtration rate: Internal and external validations of the iohexol plasma clearance technique by HPLC. Clin. Chim. Acta.

[29] Schwartz G.J., Abraham A.G., Furth S.L., Warady B.A., Muñoz A. (2010). Optimizing Iohexol Plasma Disappearance Curves to Measure the Glomerular Filtration Rate in Children with Chronic Kidney Disease. Kidney Int..

[30] Åsberg A., Bjerre A., Almaas R., Luis-Lima S., Robertsen I., Salvador C.L., Porrini E., Schwartz G.J., Hartmann A., Bergan S. (2019). Measured GFR by Utilizing Population Pharmacokinetic Methods to Determine Iohexol Clearance. Kidney Int. Rep..

[31] Bland J.M., Altman D.G. (1986). Statistical methods for assessing agreement between two methods of clinical measurement. Lancet.

[32] Lin L., Hedayat A., Wu W. (2012). Statistical Tools for Measuring Agreement.

[33] Lin L., Hedayat A., Sinha B., Yang M. (2002). Statistical methods in assessing agreement: Models, issues, and tools. J. Am. Stat. Assoc..

