# Supplementary material for: A Simplified Iohexol-Based Method to Measure Renal Function in Sheep Models of Renal Disease
Source: Biology (Basel). 2020 Aug 31;9(9):259. doi: 10.3390/biology9090259 (PMC7564881; doi:10.3390/biology9090259)
Supplement: Supplementary file 1 [file biology-09-00259-s001.pdf]

Supplementary material

1. A.- Testing of the SM in swine

To further testing the simplified two-compartment method (SM) in another species of large animals, we took advantage of a previous study of our grup in swine [39]. In brief, a single dose of 10 mL Omnipaque 300 (GE Healthcare, Madrid, Spain) containing 6.47 g iohexol was intravenously injected during 2 min in a group of Iberian swine. Then, 11 blood samples were taken at 15, 30, 45, 60, 90, 120, 180, 240, 300, 360, and 420 min, as done in sheep. Iohexol plasma concentrations were measured by HPLC–UV and the plasma clearance of iohexol calculated using one (CL1) and two-compartment (CL2) models and CL1 with a correction formula to adjust CL1 to CL2. Note that in the original study in swine the CL1 corrected by a formula was named SM. For this supplementary analysis the latter is called as CL1*f* [39].

2. Pharmacokinetic analyses previously developed in swine

In swine, the duration of the distribution phase of iohexol is 120 min, and curvilinear. The elimination phase, from 120 onwards, is linear. So, 120 min is the time-point where the clearance of iohexol changes from the first to the second exponential curve.

a.- Two-compartment model (CL2): To determine CL2, all the sampling points i.e. 15, 30, 45, 60, 90, 120, 180, 240, 300, 360, and 420 min were used, covering both the distribution and elimination phases. By using the trapezoidal rule, we determined the area under the curve (AUC) of the first exponential curve (distribution phase) from 0 to 120 ($AUC_{0–120}$) and the area under the curve of the second exponential curve (elimination phase) from 120 to 420 ($AUC_{120–420}$). Also, we estimated AUC from 420 min to infinity ($AUC_{420–inf}$) obtained by dividing the iohexol concentration at 420 min by the elimination constant determined by a log-linear regression of the concentrations from 120 to 420 min. So, we calculated the total AUC from 0 to infinity ($AUC_{0–inf}$) Finally, the plasma clearance of iohexol was determined by the formula: CL= Dose/AUC, where AUC is the area under the plasma concentration-time profile.

b.- One-compartment model (CL1): only sampling points of the second part of the curve (elimination phase) at 120, 180, 240, 300, 360, and 420 min were used. By using the trapezoidal rule, we calculated the AUC from 0 to 420 min ($AUC_{0–420}$), and then we added the area from the last sampling point (420 min) by dividing this concentration by the elimination rate constant obtained by a log-linear regression of the concentrations from 120 to 420 min. Therefore, we determined the total AUC from 0 to infinity ($AUC_{0–inf}$). The plasma clearance of iohexol was calculated by the formula: CL= Dose/AUC, where AUC is the area under the plasma concentration-time profile.

c.- One-compartment model adjusted by a correction formula (CL1f): GFR calculated by one-compartment model (CL1) overestimated true GFR assessed by CL2 (Table S1). CL1 overestimated the AUC because it does not consider the initial distribution phase of iohexol. Thus, a formula is needed to adjust CL1 to CL2 and calculate the true clearance. After analyzing several types of formulas, the more accurate equation to adjust CL1 to CL2 was the first-order polynomial quadratic equation: $CL1f = -47.909 + (1.176 \times CL1) - (0.00063968 \times CL12)$ where CL1 is the clearance obtained based on the one-compartment model and CL1*f* the recalculated true clearance with the simplified method. The mean absolute percentage error (MAPE) was 6.2%. Individual GFR values for the group of 16 pigs are shown in Table S1.

3. *Simplified two-compartment model (CL2 simplified - SM)*

To test the validity of the simplified two-compartment model (SM) developed in sheep, we evaluated this pharmacokinetic approach in a group of 16 pigs previously studied by our research group [39]. We selected 7 plasma samples covering both the distribution and elimination phases i.e. 15, 30, 90, 120, 180, 240 and 300 min, to calculate the plasma clearance of iohexol by a simplified two-compartment model. The performance of the SM was compared with the reference method (CL2), observing a very low mean absolute percentage error (MAPE) of 2.9%. This outperformed the bias observed for the one-compartment model corrected by a first-order polynomial quadratic equation (CL1*f*), that showed a MAPE of 6.2%.

Case	Gender	Weight (kg)	CL2 (ml/min)	CL1*f* (ml/min)	CL1*f*-CL2 (MAPE)	SM (ml/min)	SM–CL2 (MAPE)
1	F	176	290	271	6.6	304	4.8
2	F	113	120	115	4.2	122	1.7
3	F	106	174	174	0.0	175	0.6
4	F	122	173	194	12.1	175	1.2
5	F	209	301	282	6.3	305	1.3
6	F	188	314	293	6.7	321	2.2
7	F	115	203	213	4.9	194	4.4
8	F	116	112	92	17.9	102	8.9
9	F	138	226	249	10.2	224	0.9
10	F	182	247	228	7.7	244	1.2
11	F	212	392	394	0.5	406	3.6
12	F	159	290	317	9.3	287	1.0
13	F	132	234	211	9.8	236	0.9
14	F	156	203	209	3.0	202	0.5
15	F	210	342	341	0.3	353	3.2
16	F	101	176	177	0.6	193	9.7

Table S1. Iohexol plasma clearance in swine. Two compartment model CL2 is the reference method. CL1: one compartment model; CL1*f*: one compartment model corrected with a formula; SM: simplified two-compartment using 7 points. (MAPE = mean absolute percentage error.

4. *Analysis of agreement*

a.- CL2 vs CL1*f*

The Bland–Altman plot (Figure S1A) showed narrow limits of agreement (from −30.6 to 34.9 mL/min) and a mean difference of −2.3 mL/min between values measured with the one-compartment model with a correction formula (CL1*f*) and the reference method (CL2), indicating good agreement. The concordance correlation coefficient (CCC) was 0.97 (0.94, upper confidence interval -CI); the total deviation index (TDI) 14.7% (20.6), which means that 90% of the GFR values showed an error ranging from −14.7 to +14.7% when compared with the reference method. Finally, the coverage probability (cp) was 71 (54), which indicates that more than 29% of the GFR values had an error range greater than 10% of CL2.

a.- CL2 vs SM

The Bland–Altman plot (Figure S1B) showed very narrow limits of agreement between the two compartment models, CL2 and SM: from −12.7 to 18.5 mL/min and a mean difference of 2.9 mL/min, indicating excellent agreement. The CCC was 0.993 (0.986, upper confidence interval (CI), reflecting excellent precision and accuracy; the TDI was 6.9%, which means that 90% of the GFR values showed an error ranging from −6.9 to +6.9% when compared with the reference method; CP: 97% (84-CI), which indicates that only 3% of the GFR values had an error range greater than 10% of CL2.

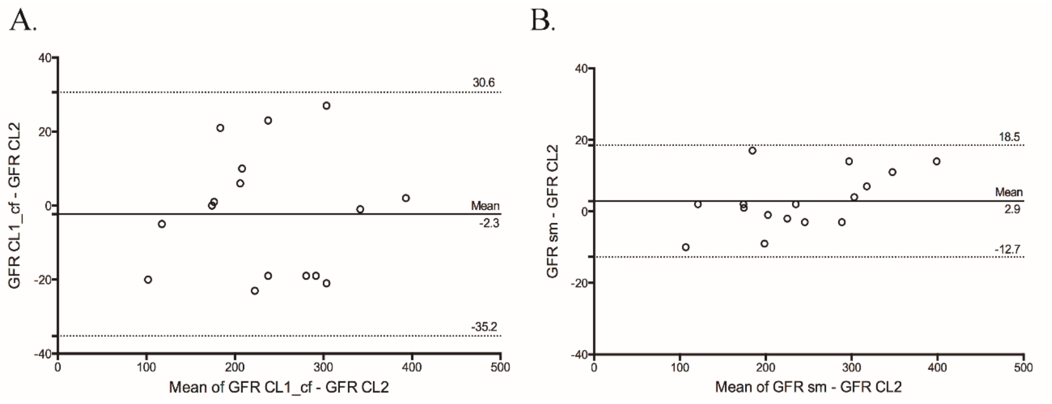

Figure S1. Bland–Altman plots of the difference between the glomerular filtration rate (GFR) values measured by the reference method (two-compartment clearance: CL2) and (**A**) the one compartment method adjusted by a formula CL1*f* and (**B**) the simplified method (SM) versus the differences of the means of both. The straight and dashed lines indicate mean difference and 95% limits of agreement, respectively.

	Total Deviation Index (%)	Concordance Correlation Coefficient (%)	Coverage Provability (%)	Limits of Agreement (mL/min)
CL1*cf*	14.7 (20.6)	0.974 (0.941)	71 (54)	from −35.2 to 30.6
SM	6.9 (9.6)	0.993 (0.986)	97 (84)	from −12.7 to 18.5

Table S2. Agreement between the two-compartment reference method (CL2) with the one-compartment model adjusted by an equation (CL1*f*) and the two-compartment simplified method (SM) in a group of 16 pigs. TDI: total deviation index, TDI (total deviation index); CCC (concordance correlation coefficient); CP (coverage probability); LA (limits of agreement, ml/min).
